# Infection, dissemination, and transmission efficiencies of Zika virus in *Aedes aegypti* after serial passage in mosquito or mammalian cell lines or alternating passage in both cell types

**DOI:** 10.1186/s13071-021-04726-1

**Published:** 2021-05-18

**Authors:** Lourdes G. Talavera-Aguilar, Reyes A. Murrieta, Sungmin Kiem, Rosa C. Cetina-Trejo, Carlos M. Baak-Baak, Gregory D. Ebel, Bradley J. Blitvich, Carlos Machain-Williams

**Affiliations:** 1 Laboratorio de Arbovirología, Centro de Investigaciones Regionales “Dr. Hideyo Noguchi”, Universidad Autónoma de Yucatán, Mérida, México; 2 Department of Microbiology, Immunology and Pathology, College of Veterinary Medicine and Biomedical Sciences, Colorado State University, Fort Collins, CO USA; 3 Department of Infectious Diseases in Internal Medicine, Sejong Chungnam National University Hospital, School of Medicine, Chungnam National University, Sejong, Korea; 4 Department of Veterinary Microbiology and Preventive Medicine, College of Veterinary Medicine, Iowa State University, Ames, IA USA

**Keywords:** Zika virus, Flavivirus, *Aedes aegypti*, Adaptive mutations, Vectorial competence

## Abstract

**Background:**

Zika virus (ZIKV) is an arthropod-borne virus (arbovirus) with an urban transmission cycle that primarily involves humans and *Aedes aegypti.* Evidence suggests that the evolution of some arboviruses is constrained by their dependency on alternating between disparate (vertebrate and invertebrate) hosts. The goals of this study are to compare the genetic changes that occur in ZIKV after serial passaging in mosquito or vertebrate cell lines or alternate passaging in both cell types and to compare the replication, dissemination, and transmission efficiencies of the cell culture-derived viruses in *Ae. aegypti*.

**Methods:**

An isolate of ZIKV originally acquired from a febrile patient in Yucatan, Mexico, was serially passaged six times in African green monkey kidney (Vero) cells or *Aedes albopictus* (C6/36) cells or both cell types by alternating passage. A colony of *Ae. aegypti* from Yucatan was established, and mosquitoes were challenged with the cell-adapted viruses. Midguts, Malpighian tubules, ovaries, salivary glands, wings/legs and saliva were collected at various times after challenge and tested for evidence of virus infection.

**Results:**

Genome sequencing revealed the presence of two non-synonymous substitutions in the premembrane and NS1 regions of the mosquito cell-adapted virus and two non-synonymous substitutions in the capsid and NS2A regions of both the vertebrate cell-adapted and alternate-passaged viruses. Additional genetic changes were identified by intrahost variant frequency analysis. Virus maintained by continuous C6/36 cell passage was significantly more infectious in *Ae. aegypti* than viruses maintained by alternating passage and consecutive Vero cell passage.

**Conclusions:**

Mosquito cell-adapted ZIKV displayed greater *in vivo* fitness in *Ae. aegypti* compared to the other viruses, indicating that obligate cycling between disparate hosts carries a fitness cost. These data increase our understanding of the factors that drive ZIKV adaptation and evolution and underscore the important need to consider the *in vivo* passage histories of flaviviruses to be evaluated in vector competence studies.

**Graphical abstract:**

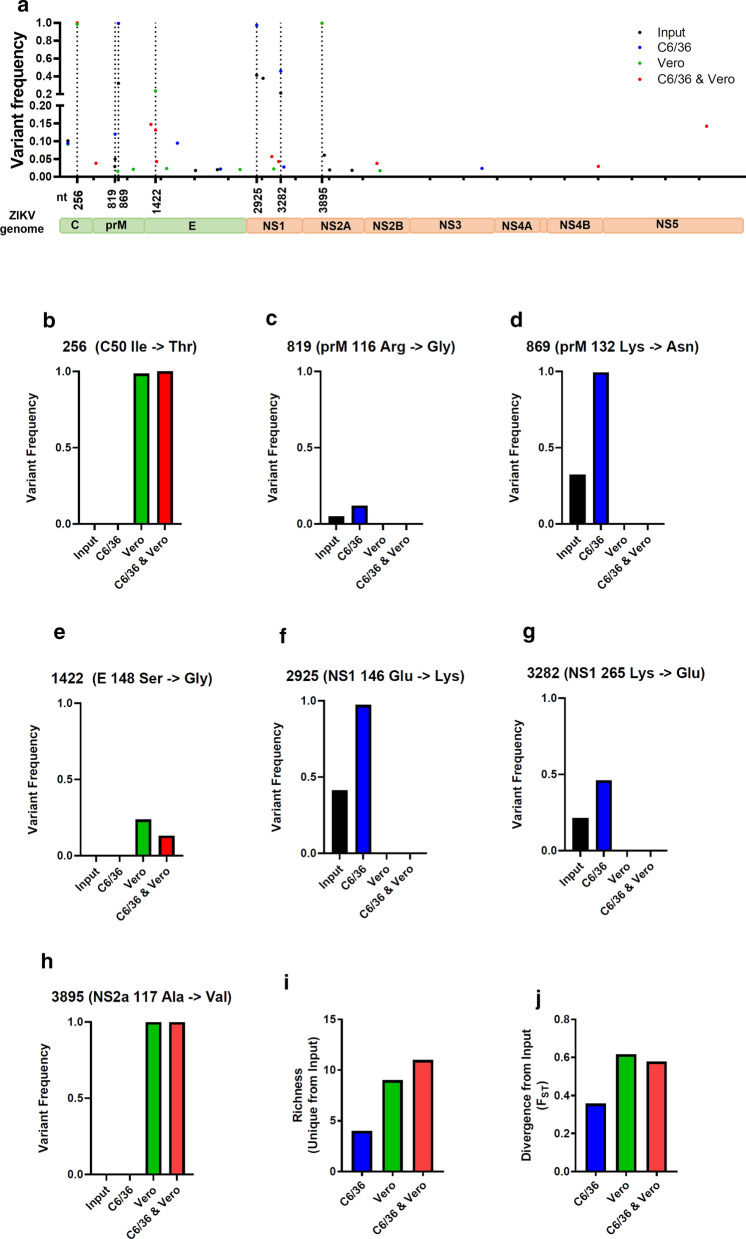

**Supplementary Information:**

The online version contains supplementary material available at 10.1186/s13071-021-04726-1.

## Background

Zika virus (ZIKV) is an arthropod-borne virus (arbovirus) that belongs to the genus *Flavivirus* (family *Flaviviridae*). ZIKV was first isolated from a febrile rhesus monkey in Zika Forest, Uganda, in 1947, with subsequent isolations made from *Aedes africanus* in the same region the following year [1]. The first known infections in humans occurred in Nigeria in 1954 [2, 3]. Afterwards, the virus was detected elsewhere in Africa and Asia, but cases of human disease were sporadic [4]. ZIKV emerged in the Pacific Islands in 2007, causing an explosive outbreak of febrile illness on Yap Island, with infections occurring in most inhabitants [5]. Additional outbreaks of ZIKV have occurred elsewhere in the Pacific Islands [6, 7]. The virus underwent a dramatic expansion of its geographic distribution in 2015, when it was reported for the first time in the Western Hemisphere [6]. ZIKV now occurs throughout the tropical Americas.

ZIKV is primarily maintained in transmission cycles involving *Aedes* spp. mosquitoes and human or nonhuman primates, although other modes of transmission (i.e. sexual transmission between humans) have been documented. The principal urban transmission vector of ZIKV in the Americas is *Aedes aegypti*, but other *Aedes* spp. mosquitoes also transmit the virus [8–10]. In humans, ZIKV infections are usually asymptomatic or self-limiting but serious manifestations, such as congenital microcephaly and Guillain-Barré syndrome in adults, can occur [11–13].

ZIKV has a positive-sense, single-stranded RNA genome. The genomes of ZIKV and most other RNA viruses encode an RNA-dependent RNA polymerase, an enzyme that incorporates a relatively high number of mutations, ranging from 10^–4^ to 10^–6^ mutations per site per round of replication, due to the lack of a proofreading exonuclease activity [14, 15]. Most mutations have no effect on viral fitness or are quickly removed from the virus population by natural selection. Some mutations, however, are advantageous and can result in enhanced virus transmission or increased disease severity, resulting in epidemics of increased severity, as documented for several arboviruses, including West Nile virus (WNV), Venezuelan equine encephalitis virus (VEEV), chikungunya virus (CHIKV) and ZIKV [16–20]. An amino acid substitution (V159A) in domain I of the envelope protein of WNV appeared in 2002 and resulted in a significantly decreased extrinsic incubation period in *Culex* mosquitoes, thereby enhancing vectorial capacity [16]. Additionally, an amino acid substitution (T249P) in the NS3 protein of a relatively attenuated strain of WNV produced a virus that was highly lethal to American crows [17]. A mutation (S218N) in the E2 protein of VEEV enhanced viral infection, replication and transmission in *Aedes taeniorhynchus*. The mutation was linked to an epizootic outbreak of VEEV in horses from Mexico in 1993–1996 [18]. In 2007, a substitution (A226V) in the E1 protein of CHIKV was responsible for significantly increased infectivity and more efficient dissemination and transmission of the virus by *Aedes albopictus*, facilitating its rapid spread [20]. Genetic changes in the viral RNA genome can therefore have a major effect on arbovirus transmission, epidemiology and disease presentation.

RNA viruses that cycle between disparate (vertebrate and invertebrate) hosts typically experience lower evolutionary rates than RNA viruses that replicate in a single host [21, 22]. Alternate host replication has been hypothesized to impose a fitness trade-off that constrains genome evolution and host-specific adaptation. Many studies have been performed to test this hypothesis and to evaluate the genotypic and phenotypic consequences of sequential and alternating host cell passaging of arboviruses [23–33]. One of the earliest studies was performed by Novella and colleagues, who reported a lack of evolutionary stasis during alternating replication of vesicular stomatitis virus in baby hamster kidney (BHK-21) and sandfly (LL-5) cell lines [29]. In another study, VEEV serially passaged in *Ae. aegypti* exhibited increased mosquito infectivity when compared to parental virus while sequential passage in mice yielded a virus that produce higher viremias [34]. Alternately passaged VEEV exhibited no apparent fitness gains in either host. The goal of this study was to determine the effects of *in vitro* host specialization on the genomic composition of ZIKV and *in vivo* replicative ability of the virus in geographically matched mosquitoes.

## Methods

### Cell lines and virus

African Green Monkey kidney (Vero) and *Aedes albopictus* (C6/36) cells were obtained from the American Type Culture Collection (Manassas, VA). Vero cells were cultured in Dulbecco’s modified Eagle medium (Invitrogen, Carlsbad, CA), and C6/36 cells were cultured in Leibovitz L15 medium (Invitrogen). All media were supplemented with 10% fetal bovine serum (FBS), 100 units/ml penicillin and 100 μg/ml streptomycin. Mammalian cells were cultured at 37 °C with 5% CO_2_ whereas C6/36 cells were cultured at 28 °C. ZIKV (strain ZIKV-Mer17) was originally isolated from a febrile patient in Merida, Yucatan, in 2017 and had been passaged four times in C6/36 cells prior to this study. The virus was titrated by plaque assay following published protocols [35], and titers were expressed as plaque-forming units per milliliter (PFU/ml).

### Serial passaging of ZIKV in cell culture

ZIKV was sequentially passaged six times in one cell type (Vero or C6/36 cells) or alternately between both cell types (Vero then C6/36 cells) in triplicate 75 cm^2^ flasks. Original cultures were inoculated with ZIKV at a multiplicity of infection (m.o.i) of 0.01, and subsequent cultures were inoculated with 100 µl of cell culture supernatant collected from the prior passage. Supernatants were harvested from Vero cells when 70–90% of the cell monolayer exhibited cytopathic effect and from C6/36 cells at 6 days post-infection. Aliquots of the triplicate supernatants harvested after the final passage were consolidated before use in the vector competence experiments.

### Mosquitoes

Immature *Ae. aegypti* were collected at field sites in 2018 in the city of Merida, Yucatan. Mosquitoes were transported to the insectaries at the Laboratorio de Arbovirología at the Universidad Autónoma de Yucatán and reared to adults. After mating, eggs were collected, dehydrated and sent to Colorado State University (CSU). Eggs were hatched and reared to adults in the insectaries at the CSU Arthropod-Borne and Infectious Diseases Laboratory. Adults were maintained with water and sucrose *ad libitum* under standard conditions (28 °C, 70–80% humidity, light-dark photoperiod of 12:12 h). To facilitate egg production, females were provided defibrinated calf blood at 37 °C using an artificial membrane and water-jacketed glass feeder. All experimental infections were performed using F3 adults.

### Vector competence

Infectious blood meals were prepared by mixing virus (1 × 10^6^ PFU/ml) and defibrinated calf blood at a ratio of 1:1. Blood meals were offered for 30 min to 5–9 day-old *Ae. aegypti* using a water-jacketed feeder and hog gut membrane. The mosquitoes were deprived of sucrose and water for 24 h prior to viral infection. Mosquitoes were cold-anesthetized, and engorged females were selected and held in the insectary. At 3, 5, 7, 14 and 21 days post-infection (dpi), 27–30 females were selected from each group and cold-anesthetized. Wings and legs were removed and placed into 2-ml tubes containing 200 μl of diluent and one stainless steel bead. The diluent consisted of phosphate buffer saline (PBS), pH 7.4, supplemented with 20% FBS, antibiotics and fungizone. Once the wings and legs were removed, saliva was obtained from each female as previously described by Smith et al. in 2005 [36]. Briefly, the proboscis was placed into a capillary tube containing immersion oil, allowing females to expectorate in the tube. After 30 min, the capillary tube was broken into a conical tube containing 100 μl of diluent. Once salivation was complete, midguts, Malpighian tubules, ovaries, and salivary glands were harvested. Tissues were rinsed individually in a drop of PBS and placed in separate tubes containing 200 μl of diluent and a stainless steel bead. Tissue homogenization was carried out at 24 Hz for 5 min. Tubes containing homogenates and saliva were centrifuged at 20,000× g for 5 min and stored at − 80 °C. These experiments were performed in duplicate in the Biosafety Level 3 facilities at CSU.

Midgut infection rates (MIRs), dissemination infection rates (DIRs), salivary gland infection rates (SGIRs), and transmission rates (TRs) were calculated and compared between viruses. MIR was defined as the number of mosquitoes with infectious ZIKV in their midguts as determined by plaque assay divided by the total number of mosquitoes examined multiplied by 100. DIR was defined as the number of mosquitoes with ZIKV in non-midgut tissues (e.g. wings/legs, salivary glands, Malpighian tubules or ovaries) divided by the number of mosquitoes with ZIKV in their midguts multiplied by 100. SGIR was defined as the number of mosquitoes with ZIKV in their salivary glands divided by the number of mosquitoes with ZIKV-infected midguts multiplied by 100. TR was defined as the number of mosquitoes with ZIKV in their saliva divided by the total number of mosquitoes with disseminated infections multiplied by 100. DIRs were often not determined if no more than five of the mosquitoes in the group had midgut infections. Likewise, SGIRs and TRs were often not determined if no more than five of the mosquitoes in the group contained virus in non-midgut tissues and their salivary glands, respectively. Due to the large number of tissues tested and, more importantly, the small size of select tissues (particularly the salivary glands), it was not feasible to perform duplicate serial dilutions of each tissue homogenate for the plaque assay analysis in order to calculate virus titers. Instead, plaque assay data were recorded as positive or negative.

### Quantitative RT-PCR

Total RNA was extracted from cell culture supernatants using the Mag-Bind® Viral DNA/RNA 96 kit (Omega Bio-Tek) on the KingFisher Flex Magnetic Particle processor (Thermo Fisher Scientific, Waltham WA) and assayed for ZIKV RNA by quantitative reverse transcriptase-polymerase chain reaction (qRT-PCR). Full-length ZIKV RNA generated from a plasmid using T7 polymerase and diluted tenfold (10^8^ to 10^2^ ng/µl) to create a standard curve, which was used to calculate viral RNA copy numbers in select mosquito organs. Reactions were performed using SsoAdvanced Universal SYBR Green supermix (Bio-Rad Laboratories, Hercules, CA) and ZIKV-reactive primers that amplify a region of the NS5 gene and 3′ untranslated region [37]. The QuantStudio 3 Real-Time PCR system (Applied Biosystems, Foster City CA) was used, with amplifying conditions of 50 °C for 15 min, 95 °C for 60 s, followed by 40 cycles of 95 °C for 15 s and 60 °C for 30 s.

### Next-generation sequencing

Viral RNA was prepared for next-generation sequencing using the Nextera XT library preparation kit (New England BioLabs, Ipswich, MA) following the manufacturer’s instructions. Briefly, viral RNA was quantified, with amounts expressed as genome equivalents (GE/ml), treated with RQ1 DNase (Promega) and used as templates in reverse transcription reactions performed using Superscript IV (ThermoFisher) and random 15-mer primers. Reaction products were treated with RNase H and amplification using the NEBNext® Ultra™ II Q5® Master Mix (New England Biolabs) and random 15-mer primers. Amplified products were fragmented and adapters and indexes added using Nextera XT (Ilumina) with in house Nextera XT dual indexes per manufacturer protocol. The index libraries were then amplified with a KAPA Real-time Library Amplification Kit (KAPA BioSystems, Indianapolis, IN) and purified using Ampure XP beads (Beckman). Final libraries were pooled by Qubit (Invitrogen) concentration and analyzed for size distribution using the Agilent High Sensitivity D1000 Screen Tape on an Agilent Tapestation 2200. Final quantification was performed using the NEBNext® Library Quant Kit for Illumina® (New England Biolabs), and 300 nt pair-end reads were generated using the Illumina MiSeq at the CSU Next-Generation Sequencing Facility.

### Bioinformatics

Next-generation sequencing data were analyzed using a custom Snakemake [38] workflow, designated as “RNA virus Population Genetics (RPG) Workflow.” Briefly, Read 1 and Read 2.fastq files from paired-end Illumina MiSeq data were trimmed to remove adaptor sequences and quality trimming of phred scores < 30 from the 3′ and 5′ read ends using cutadapt [39]. Reads were then mapped to the ZIKV-I reference sequence using MOSAIK as previously described [40, 41]. Picard (BroadInstitute), Genome Analysis Toolkit (GATK) (BroadInstitute), and SAMtools [42] were used for variant calling preprocessing. Single-nucleotide variants (SNVs), insertions and deletions (INDELS) were called using LoFreq with the –call-indels command; otherwise, default settings were used [39]. Consensus sequences were generated using the.vcf files generated above and VCFtools [43].

### Genetic diversity

Markers of genetic diversity were assessed using the RPG Workflow analysis scripts. Richness was calculated by the sum of the unique (not found in the virus input ZIKV-I population) intrahost single-nucleotide variant (iSNV) sites detected in the coding sequence (CDS) in each population. *F*_ST_ was used to estimate genetic divergence between two viral populations as described by Fumagalli et al. [44]:

1 $$a_{s} = \frac{{4n_{i} (p_{i,s} - p_{s} )^{2} + 4n_{j} (p_{j,s} - p_{s} )^{2} - b_{s} }}{{2\left( {\frac{{2n_{i} n_{j} }}{{n_{i} + n_{j} }}} \right)}},$$

and

2 $$b_{s} = \frac{{n_{i} \left( {2p_{i,s} \left( {1 - p_{i,s} } \right) + n_{j} \left( {2p_{j,s} \left( {1 - p_{j,s} } \right)} \right)} \right)}}{{n_{i} + n_{j} - 1}},$$

where *p*_*i*,*s*_, *p*_*j*,*s*_ and *p*_*s*_ are the frequencies of the input ZIKV consensus nucleotide at sites from populations *i*, *j*, and combined, respectively. Only LoFreq iSNVs were used, all other sites *p* = 1. The estimate of *F*_ST_ for the CDS of m sites is:

3 $$F_{{{\text{ST}}}}^{{\left( {{\text{locus}}} \right)}} = \frac{{\mathop \sum \nolimits_{s = 1}^{m} a_{s} }}{{\mathop \sum \nolimits_{s = 1}^{m} \left( {a_{s} + b_{s} } \right)}}.$$

### Statistical analysis

MIRs, DIRs, SGIRs and TRs were converted to the square root of the arcsine and analyzed by Kruskal-Wallis test and Mann-Whitney U-test. Differences were considered significant when *P* ≤ 0.05. Comparisons of RNA copy numbers among viruses, organs and days post-infection were performed by Friedman test. The Kruskal-Wallis test and Mann-Whitney U-test were used to compare RNA copy numbers in different organs collected on the same day post-infection. Data were transformed to log10, and results were considered significant when significance was < 5%. The statistical analyses were performed using SPSS version 22 (IBM Corporation Armonk, NY). ZIKV-6V and ZIKV-6A were not evaluated because a small number of mosquitoes tested positive for these viruses, prohibiting a robust statistical analysis.

## Results

### Genetic characterization of the host-cell adapted viruses

ZIKV was sequentially passaged six times in Vero or C6/36 cells or alternately between both cell types, and the viruses harvested from the final cultures were designated as ZIKV-6V, ZIKV-6C and ZIKV-6A, respectively. The genomes of the input virus (designated ZIKV-I) and viruses collected after the final passage were sequenced (Genbank Accession Nos. MT507047 to MT507050). ZIKV-6C contained two non-synonymous substitutions when compared to ZIKV-I, and these were located in the prM and NS1 protein genes (Table 1). The genome sequences of ZIKV-6V and ZIKV-6A were identical to each other and both contained two non-synonymous substitutions when compared to ZIKV-I. These substitutions were located in the capsid (C) and NS2A protein genes. The estimated reads per sample was between 600,000 and 2,500,000 with an average coding of 5621 (ZIKV-0), 6926 (ZIKV-1), 16110 (ZIKV-2), and 1276 (ZIKV-3) for each nucleotide position.

**Table 1:** Non-synonymous mutations identified in the genomes of Zika virus after six passages in cell culture

Virus passage history	Nucleotide position	Codon sequence^a^	Amino acid position	Amino acid
ZIKV-I	256	ATT	C 50	Ile
ZIKV-6C		ATT		Ile
ZIKV-6V		A**C**T		Thr
ZIKV-6A		A**C**T		Thr
ZIKV-I	869	AAA	prM 132	Lys
ZIKV-6C		AA**C**		Asn
ZIKV-6V		AAA		Lys
ZIKV-6A		AAA		Lys
ZIKV-I	2925	GAA	NS1 146	Glu
ZIKV-6C		**A**AA		Lys
ZIKV-6V		GAA		Glu
ZIKV-6A		GAA		Glu
ZIKV-I	3895	GCG	NS2A 117	Ala
ZIKV-6C		GCG		Ala
ZIKV-6V		G**T**G		Val
ZIKV-6A		G**T**G		Val

^a^Nucleotide substitutions are bolded

### Intrahost genetic diversity

**Fig. 1:**
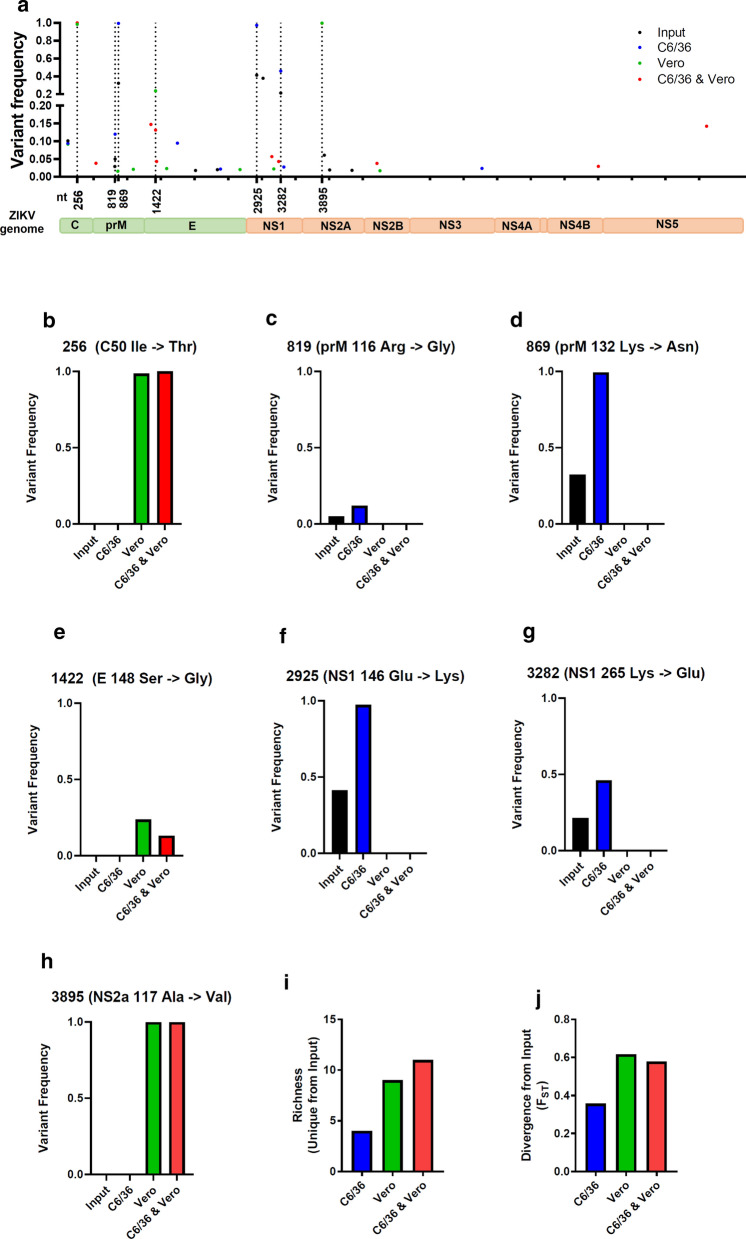
Intrahost variant frequency analysis of strains tested. Intrahost variant (iSNVs) frequency analysis of four strain tested. distributed across the ZIKV CDS (**a**). iSNV frequency analysis of passage-specific mutations (**b**–**h**). The sum of novel variants not found in ZIKV-6C, ZIKV-6V and ZIKV-6A, which are unique to those observed in ZIKV-I (**i**). Genetic divergence (*F*_ST_) of ZIKV-6C, ZIKV-6V and ZIKV-6A was compared with ZIKV-I (**j**)

An intrahost frequency analysis was performed on ZIKV-I and the three final passage viruses (Fig. 1a). Standing variation was detected in ZIKV-I, with 12 iSNVs identified, mostly in the 5′ half of the genome. Four iSNVs identified in ZIKV-6C were absent from viruses passaged in Vero cells and were located at prM-R116G, prM-K132N, NS1-E146K and NS1-K265E (Fig. 1c, d, f, g). prM-K132N and NS1-E146K became fixed. Two sSNVs unique to viruses passaged in Vero cells (ZIKV-6V and ZIKV-6A) also became fixed and were located at capsid-I50T and NS2A-A117V (Figs. 1b and 1h). The richness of ZIKV-6C, ZIKV-6V and ZIKV-6A (Fig. 1i) and their divergence from ZIKV-I (Fig. 1j) was also assessed. Novel variants accumulated more often when passaged exclusively on Vero cells (*n* = 9) or by alternating between both cell types (*n* = 11). The fixation index (*F*_ST_) of ZIKV-6C, ZIKV-6V and ZIKV-6A were estimated and compared to the *F*_ST_ of ZIKV-I (Fig. 1j). ZIKV-6V and ZIKV-6A diverged from ZIKV-I to a similar degree, with *F*_ST_ values of 0.62 and 0.58 respectively, while ZIKV-6C diverged less (*F*_ST_ = 0.36). No iSNVs were identified in the 5′ and 3′ UTRs.

### Comparison of infection, dissemination and transmission rates

**Fig. 2:**
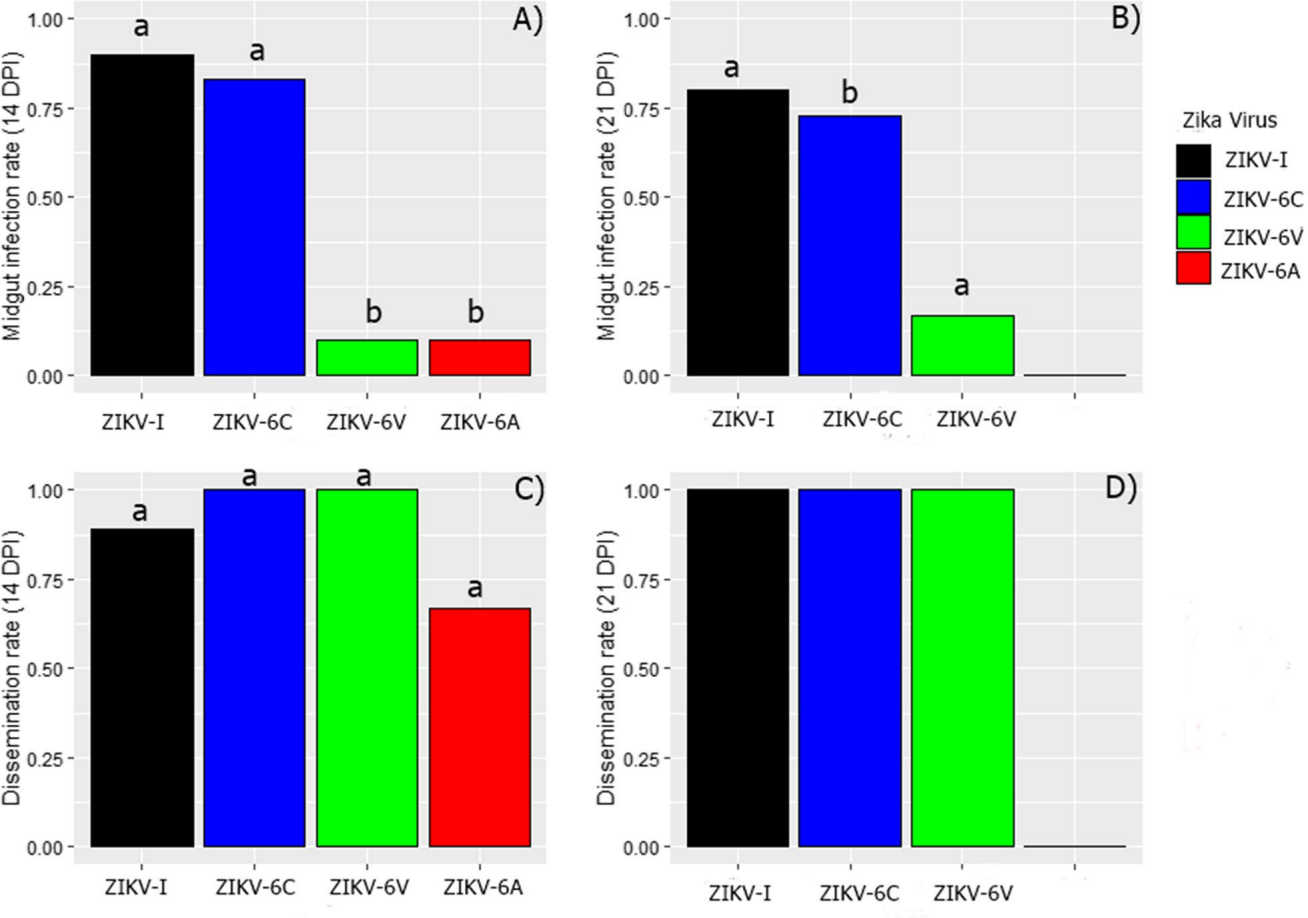
Midgut infection and dissemination rates of *Ae. Aegypti* infected with host-cell adapted Zika virus. Midgut infection rates of *Ae. aegytpi* infected with host cell-adapted Zika virus at (**a**) 14 days and (**b**) 21 days after infectious blood meal (Kruskal-Wallis test, *X*^2^ = 70.288, df = 3, *P* ≤ 0.05 and *X*^2^ = 58.807, df = 3, *P* ≤ 0.05, respectively). Dissemination rates to organs other than midgut of *Ae. aegytpi* challenged with host cell-adapted Zika virus at **c** 14 days (Kruskal-Wallis test, *X*^2^ = 3.280, df = 2, *P* ≥ 0.05) and **d** 21 days after infectious blood meal (not statistically proven because all three strains have a ratio of 1). Bars with a different letter are statistically significant

Select organs and saliva were collected from 1148 *Ae. aegypti* females that had been orally challenged with ZIKV and were tested for virus by plaque assay. These data were used to calculate midgut infection, salivary gland infection, dissemination and transmission rates (Table 2). MIRs reached as high as 0.97 and 0.83 for mosquitoes infected with ZIKV-I and ZIKV-6C, respectively. In contrast, the MIRs for mosquitoes infected with ZIKV-6V and ZIKV-6A were considerably lower and never exceeded 0.27 and 0.10, respectively. The results were analyzed by Kruskal-Wallis test, revealing that the MIRs for ZIKV-I and ZIKV-6C were significantly higher than ZIKV-6V at the end of the 21 dpi (*X*^2^ = 13.574, df = 3, *P* ≤ 0.05; Fig. 2). The MIR for ZIKV-6A at 21 dpi was not calculated because the MIRs for the earlier times were low.

**Table 2:** MIR, DR, SGIR and TR for *Ae. aegypti* mosquitoes infected with host cell-adapted ZIKV generated

Strain	Rate	Days post-infection				
		3	5	7	14	21
		Percentage of infected mosquitoes (number infected/number tested)				
ZIKV-I	MIR	97 (29/30)	70 (21/30)	77 (23/30)	90 (27/30)	80 (24/30)
	DIR	14 (4/29)	14 (3/21)	39 (9/23)	89 (24/27)	100 (24/24)
	SGIR	NT	5 (1/21)	9 (2/23)	89 (24/27)	96 (23/24)
	TR	NT	NT	11 (1/9)	17 (4/24)	4 (1/24)
ZIKV-6C	MIR	67 (20/30)	70 (21/30)	83 (25/30)	83 (25/30)	73 (22/30)
	DIR	15 (3/20)	10 (2/21)	40 (10/25)	100 (25/25)	100 (22/22)
	SGIR	NT	5 (1/21)	20 (5/25)	84 (21/25)	86 (19/22)
	TR	NT	NT	NT	4 (1/25)	5 (1/22)
ZIKV-6V	MIR	27 (8/30)	13 (4/30)	3 (1/30)	1 (3/30)	17 (5/29)
	DIR	NT	NT	NT	100 (3/3)	100 (5/5)
	SGIR	NT	NT	NT	66 (2/3)	100 (5/5)
	TR	NT	NT	NT	NT	NT
ZIKV-6A	MIR	4 (1/28)	7 (2/30)	3 (1/30)	10 (3/30)	NT
	DIR	NT	NT	NT	67 (2/3)	NT
	SGIR	NT	NT	NT	67 (2/3)	NT
	TR	NT	NT	NT	NT	NT

MIR: midgut infection rate (as number of mosquitoes with ZIKV-positive midguts/number of mosquitoes tested × 100); DIR: dissemination rate (number of mosquitoes with ZIKV in any organ other than the midgut/number of mosquitoes with ZIKV-positive midguts × 100); SGIR: salivary gland infection rate (number of mosquitoes with ZIKV-positive salivary gland/number of mosquitoes with ZIKV-positive midguts × 100); TR: transmission rate (number of mosquitoes with ZIKV-positive saliva/number of mosquitoes with disseminated infections × 100); NT, not tested. DIRs were usually not determined if no more than five of the mosquitoes in the group had midgut infections. Likewise, SGIRs and TRs were usually not determined if no more than five of the mosquitoes in the group contained virus in non-midgut tissues and their salivary glands, respectively. Tissues collected from the ZIKV-6A challenged mosquitoes at 21 dpi were not tested because of the small number of mosquitoes (≤ 3) that had midgut infections on 3 to 14 dpi

Disseminated infections were detected in all mosquitoes with midguts infected with ZIKV-I, ZIKV-6C and ZIKV-6V at 21 dpi; therefore, the DIRs were calculated as 1.0 (Table 1). Successful dissemination of all viruses occurred at 14 dpi, with ZIKV-I, ZIKV-6C, ZIKV-6V and ZIKV-6A yielding DIRs of 0.89, 1.0, 1.0 and 0.67, respectively. Kruskal-Wallis test revealed that there were no statistical differences in DIRs between the different groups of mosquitoes at 14 and 21 dpi (*X*^2^ = 3.280, df = 2, *P* ≥ 0.05).

**Fig. 3:**
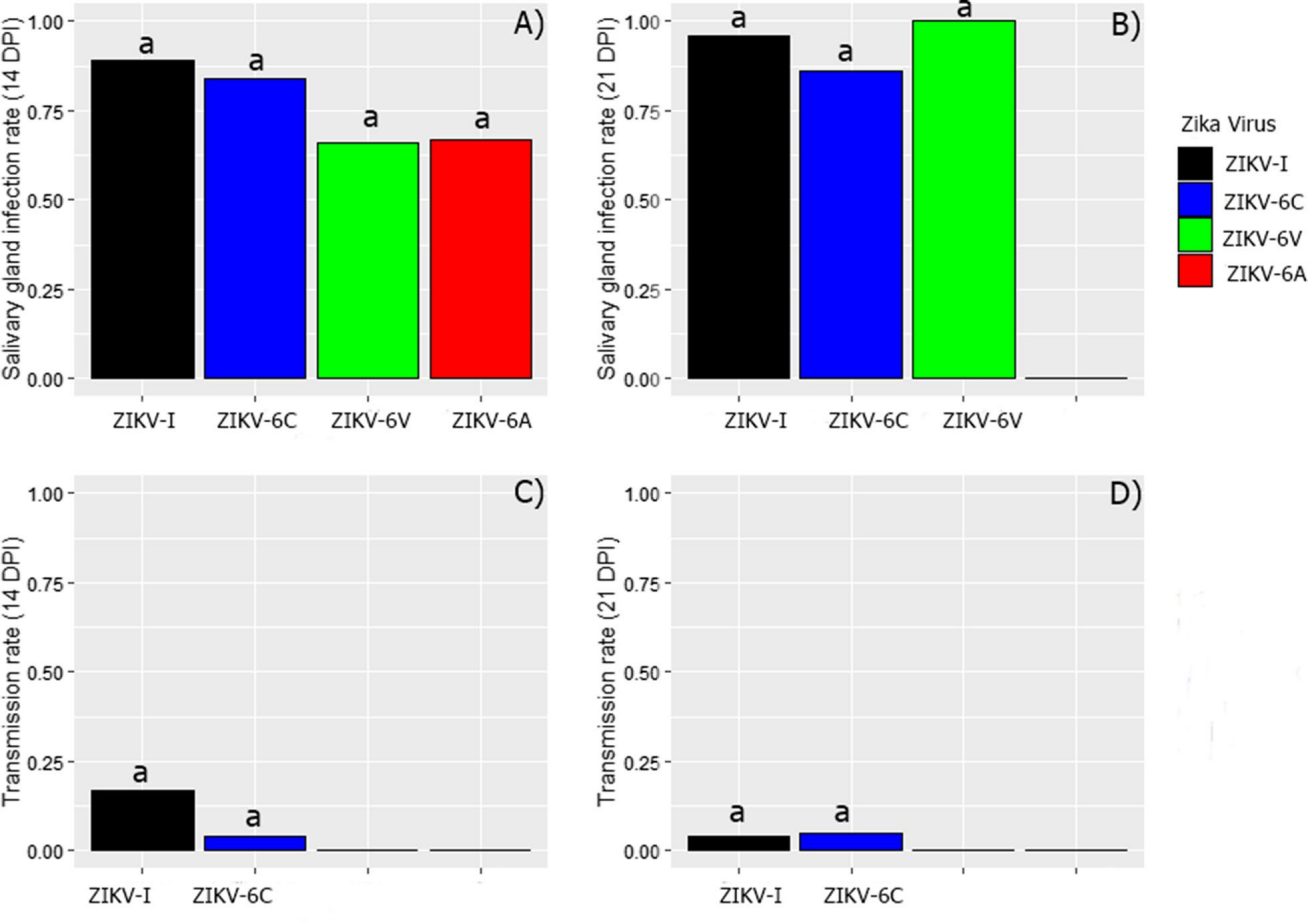
Salivary gland infection and transmission rates of *Ae. aegytpi* infected with host cell-adapted Zika virus. Salivary glands infection rates of *Ae. aegytpi* challenged with host cell-adapted Zika virus at **a** 14 days and **b** 21 days after infectious blood meal (Kruskal-Wallis test, *X*^2^ = 1.129, df = 3, *P* ≥ 0.05 and *X*^2^ = 1.858, df = 2, *P* ≥ 0.05, respectively). Transmission rates of *Ae. aegypti* challenged with host cell-adapted Zika virus at **c** 14 days and **d** 21 days after infectious blood meal (Mann-Whitney U-test, *Z* = 1.449, *P* ≥ 0.05 and *Z* = − 0.062, *P* ≥ 0.05, respectively). Bars with the same letter are not statistically significant

SGIRs as high as 0.95, 0.86, 1.0 and 0.66 were reported for mosquitoes infected with ZIKV-I, ZIKV-6C, ZIKV-6V and ZIKV-6A, respectively (Table 1). The SGIRs for the different groups of mosquitoes were not significantly different at 14 and 21 dpi (*X*^2^ = 1.129, df = 3, *P* ≥ 0.05 and *X*^2^ = 1.858, df = 2, *P* ≥ 0.05, respectively; Fig. 3).

Most saliva samples did not contain detectable virus, with TRs not exceeding 0.17 and 0.05 for mosquitoes challenged with ZIKV-I and ZIKV-6C, respectively (Table 1). Mann-Whitney U-test revealed that there were no significance differences in the TRs for these two groups of mosquitoes at 14 and 21 dpi (*Z* = 1.449, *P* ≥ 0.05 and *Z* = − 0.062, *P* ≥ 0.05, respectively; Fig. 3). Saliva from mosquitoes infected with the other two viruses were not tested because of the low numbers of mosquitoes (no more than five) that had salivary gland infections, preventing a reliable statistical analysis from being performed.

### Viral RNA copy numbers in *Ae. aegypti* organs

**Fig. 4:**
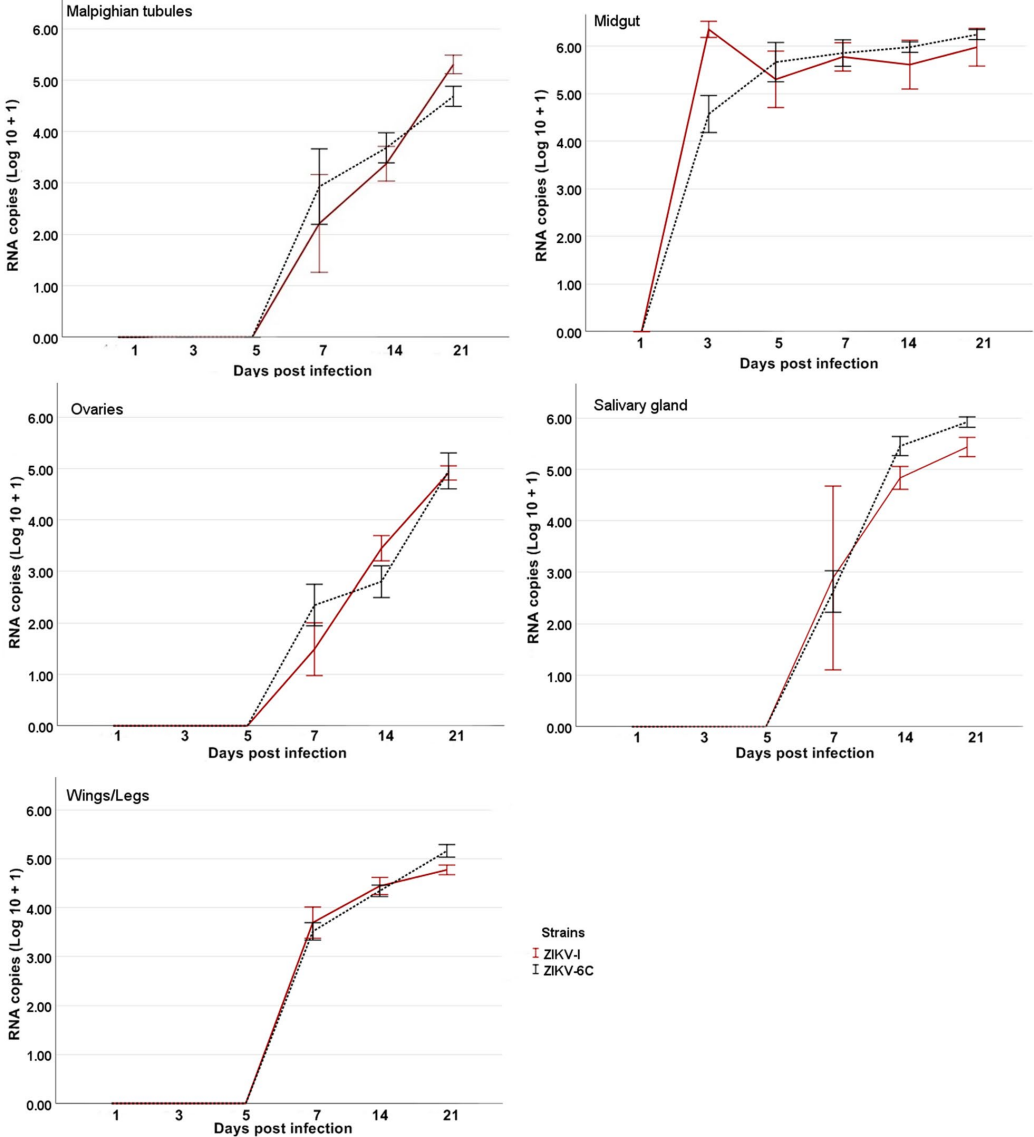
RNA copy numbers in organs and body parts from mosquitoes challenged with ZIKV-I and ZIKV-6C. The study was performed using: **a** Malpighian tubules, **b** midguts, **c** ovaries, **d** salivary glands and **e** wings/legs. The amount of viral RNA in each organ over time was determined by Friedman test. The amount of viral RNA in different organs was compared at the same time point using Mann-Whitney U and Kruskal-Wallis tests. *P* ≤ 0.05 was considered significant. ZIKV-6V and ZIKV-6A were not included in the analysis because a small number of mosquitoes tested positive for these viruses, preventing a robust statistical analysis

Total RNA was extracted from select organs and other body parts of *Ae. aegypti* that had been infected with ZIKV-I or ZIKV-6C an then tested by quantitative RT-PCR to measure the amounts of genomic RNA. Evidence of infection was detected in all organs and other body parts (midguts, ovaries, wings/legs, salivary glands, and Malpighian tubules) tested (Fig. 4). Mosquitoes infected with ZIKV-6V and ZIKV-6A were not tested because the viruses rarely infected organs other than the midgut. Likewise, no saliva samples were tested by quantitative RT-PCR because most did not contain detectable amounts of infectious virus.

For mosquitoes infected with ZIKV-I, there was a significant statistical difference in viral RNA copy numbers in midguts at 3 to 21 dpi, with copies numbers highest at the first and last time points (See Additional file 1: Table S1). There was also a significant difference in viral RNA copy numbers in Malpighian tubules, ovaries, and wings/legs at 7 to 21 dpi, with copy numbers highest at the final time point. In salivary glands, there was a significant difference in viral RNA copy numbers at 14 and 21 dpi, with copy numbers highest at the final time point. Similar findings were observed in mosquitoes infected with ZIKV-6C, with a significant statistical difference in viral RNA copy numbers in the midguts and all other body parts at 3 to 21 and 7 to 21 dpi, respectively (See Additional file 1: Table S2). On each occasion, copy numbers were highest at 21 dpi.

Viral RNA copy numbers were compared according to body part and time in mosquitoes infected with ZIKV-I and ZIKV-6C (Fig. 4). Significantly higher amounts of genomic RNA were detected in the ZIKV-I group on several occasions: midguts at 3 dpi (*Z* = − 5.723, *P* = 0.000), ovaries at 14 dpi (*Z* = − 2.103, *P* = 0.035) and Malpighian tubules at 21 dpi (*Z* = − 3.070, *P* = 0.002). Significantly higher amounts of genomic RNA were also detected in the ZIKV-6C group on several occasions: salivary glands at 14 dpi (*Z* = − 2.639, *P* = 0.008), salivary glands at 21 dpi (*Z* = − 3.085, *P* = 0.002) and wings/legs at 21 dpi (*Z* = − 2.103, *P* = 0.035).

## Discussion

RNA viruses have faster evolutionary rates than DNA viruses because they have shorter replication times and larger population sizes and their RNA-dependent RNA polymerases are error-prone and lack proofreading activity [15]. However, RNA viruses that alternate between disparate (vertebrate and invertebrate) hosts typically experience lower rates of evolution than RNA viruses that replicate in a single host [21, 22]. It has been hypothesized that alternate host replication imposes a fitness trade-off that constrains genome evolution and host-specific adaptation. Here, we provide evidence that ZIKV adopts a compromised fitness level for alternate replication in vertebrate and mosquito cells. When ZIKV was released from alternate host cell replication by sequential *in vitro* passaging, the mosquito cell-adapted virus exhibited similar *in vivo* fitness in its principal mosquito vector compared to the input virus and greater *in vivo* fitness than the alternate-passaged virus. ZIKV passaged only in Vero cells were also released from alternate host cell replication. The vertebrate cell-adapted virus exhibited lower *in vivo* fitness in *Ae. aegypti* compared to both the input virus as the virus sequentially passaged in mosquito cell cultures. Others have also shown diminished arbovirus replication in mosquito cells or mosquitoes after sequential vertebrate cell passage [28, 34, 45]. For example, sequential passage of VEEV in BHK-21 cells yielded virus that exhibited significantly reduced *in vivo* fitness in both mosquitoes and vertebrates compared to both the parental (input) virus and the virus maintained by alternating cell culture passages [34]. In contrast, VEEV sequentially passaged in mice produced significantly higher viremias compared to the parental virus as well as virus maintained by alternating passages in mice and *Ae. aegypti*, underscoring the limitations of *in vitro* systems. Our study is the first to investigate how sequential and alternating host passaging effects on the *in vivo* fitness of ZIKV in mosquitoes.

Several other studies have examined the fitness implications of releasing flaviviruses from alternate host passaging [23–28]. Serial passaging of WNV and St. Louis encephalitis virus (SLEV) in C6/36 cells increased the relative fitness and replicative ability of each virus in the mosquito cell line, but not an avian cell line [24]. Subsequent *in vivo* experiments revealed that serial passaging of WNV and SLEV in *Culex pipiens* increased the replicative ability of WNV, but not SLEV, in this mosquito species [25, 26]. The consequences of releasing arboviruses from alternate host cycling has also been studied using viruses in the families *Phenuiviridae*, *Rhabdoviridae* and *Togaviridae* [29–33]. For example, eastern equine encephalitis virus passaged exclusively in BHK or C6/36 cells exhibited reduced fitness in the bypassed cell line but replicated no more efficiently in the non-bypassed cell line compared to the alternate-passaged virus [30]. Although other studies have compared the replicative abilities of host-cell adapted and alternate-passaged arboviruses, there are several unique aspects to our study. We are the first to investigate the *in vivo* fitness implications of releasing an *Aedes*-borne flavivirus from alternate host cycling. Intrahost frequency analysis has rarely been used to determine the genetic composition of a flavivirus released from alternate host passaging [41, 46]. Additionally, geographically matched virus and *Aedes* mosquitoes were used, with the isolate having a low-passage history and the mosquitoes having been maintained in the insectary for only a few generations to minimize the effects of laboratory adaptation.

Full-genome sequencing identified two nonsynonymous substitutions in ZIKV after serial passage in C6/36 cells when compared to the input virus. One substitution (NS1-E146K) was conservative, and the other (prM-K132N) was non-conservative. Infection, dissemination and transmission rates were not significantly different in *Ae. aegypti* challenged with ZIKV-6C when compared to ZIKV-I. Our findings indicate that these substitutions do not have a favorable or detrimental effect on viral fitness in mosquitoes. Another, albeit less likely, explanation is that one substitution could be deleterious while the other is advantageous, but together they function as compensatory mutations with a net neutral fitness compared to the initial strain [47]. Compensatory mutations have previously been identified in other flaviviruses, including dengue, Japanese encephalitis and yellow fever viruses [48–50]. Two additional mutations were detected in ZIKV-6C (prM-R116G and NS1-K265E) by intrahost analysis but neither had become fixed, although the frequency of each increased after passages.

Serial passaging of ZIKV in Vero cells yielded two fixed, nonsynonymous substitutions. One substitution (NS2A-A117V) was conservative, and the other (C-I50T) was non-conservative. Both substitutions were also detected in the virus generated by six alternating passages. These mutations significantly decreased ZIKV fitness in *Ae. aegypti*. A non-fixed variant (E-S148G) that increased in frequency after cell culture passages was also identified in both the vertebrate cell-adapted and alternate-passaged viruses. The NS2A-A117V substitution has previously been detected in ZIKV [51]. Site-directed mutagenesis and *in vivo* experimental infection studies revealed that this single change enhances ZIKV virulence in mice. This change also reduces the host innate immune responses and delays the induction of apoptosis in ZIKV-infected human lung adenocarcinoma (A549) cells. The effects, if any, of the substitutions that occurred in the capsid and envelope proteins are not known. The capsid protein is required for the assembly of the nucleocapsid that surrounds and protects the flavivirus genome, but also has many other roles including apoptosis induction, lipid droplet binding, functioning as an RNA chaperone and suppression of anti-viral RNA silencing [52–54]. The envelope protein is the main structural component of flavivirus particle and participates in key steps during the viral life cycle, such as virus-mediated membrane fusion, receptor recognition and entry in susceptible host cells [55]. Site-directed mutagenesis and *in vivo* experimental infection studies would be required to determine the potential effect of these mutations on the vector competence of *Ae. aegypti* for ZIKV.

Infection and dissemination rates of ZIKV-6A in *Ae. aegypti* were low, even though the virus was maintained by alternate passaging in vertebrate and mosquito cells. This finding was not unexpected because several studies have shown large variations in the vector competence of *Ae. aegypti* from Mexico for ZIKV [56–58]. For example, Garcia-Luna and colleagues reported transmission rates of 8 to 51% using *Ae. aegypti* collected from different geographic regions in Mexico [56]. Flavivirus susceptibility in *Ae. aegypti* is dependent on genetic differences and gene flow between mosquito populations and environmental factors [59–61]. *Ae. aegypti* from Mexico are more susceptible to infection with ZIKV isolates from the African genotype than the Asian/American genotype [57]. A notable genetic difference between isolates from these genotypes is that all from the Asian/American group encode an N-linked glycosylation site in the envelope protein at E-154N while most from the African group do not [62, 63]. This glycosylation site has been linked to increased ZIKV pathogenesis in mice and implicated in the occurrence of complications in patients infected during recent outbreaks [63–65].

The vector competence experiments, which were performed using temporally and spatially matched mosquitoes and virus, revealed that our mosquitoes were inefficient vectors of ZIKV, with TRs of no more than 17%. Other vector competence studies performed using *Ae. aegypti* and ZIKV strains from the Western Hemisphere have reported TRs exceeding 50% [56, 66–69]. Although vector competence may vary according to virus dose and the route of inoculation, some of the aforementioned studies used similar doses and the same inoculation route as used in our study. Low TRs have also been documented. For example, in a study where *Ae. aegypti* from Brazil, the USA and the Dominican Republic were orally challenged with a ZIKV isolate from Mexico, TRs of 0%, 0% and 20% were reported [70]. Garcia-Luna and colleagues revealed considerable variation in the vector competence of *Ae. aegypti* from different geographic locations within Mexico, with TRs of 14 to 52% at 14 days post-blood meal [56]. Variations in vector competence could also be attributed to differences in the microbiomes and viromes of mosquito populations [71, 72]. Another consideration is that our tissue homogenates were tested by plaque assay, which has a lower limit of detection than PCR-based assays but has the advantage of specifically detecting infectious virus.

There are several limitations of the study. First, the *in vitro* experiments were performed using C6/36 cells when it would have been preferable to use a cell line derived from *Ae. aegypti* because it is the principal urban vector and, more importantly, it would have provided consistency in the mosquito species used for the cell culture and vector competence work. Nevertheless, we provide compelling evidence that cell culture history can affect the outcome of vector competence experiments. It is also important to note that *Ae. albopictus* is also a competent vector of ZIKV in urban settings and that many other *Aedes* spp. mosquitoes collected in sylvatic environments have tested positive for the virus [9, 73]. Another consideration is that the mosquito and vertebrate cell lines were maintained at different temperatures (28 °C and 37 °C, respectively), which could have selected for genetic changes in the virus. The inclusion of *in vitro* experiments where both cell types are maintained at the same temperature (i.e. 32 °C) as done by others would have identified temperature-specific mutations [28, 30]. Viruses collected after each cell culture passage were not titrated at each passage were not considered, and founding populations were not taken into consideration, although we estimate that the cells were inoculated with virus at MOIs well below 0.1 and that there were one or two generations per passage. The study could also have been strengthened by evaluating the vector competence of *Ae. aegypti* for the viruses collected after every cell culture passage but, due to logistical constraints, it was not feasible to challenge and process the number of mosquitoes needed to complete this work. The absence of *in vitro* fitness assays is another limitation, and a more robust analysis could have been performed if more than six cell culture passages were used. Viruses were not sequenced after each cell culture passage. Likewise, viruses in mosquito tissues were not sequenced, and the inclusion of these experiments would have identified reversions as well as other mutations that could have affected vector competence. Additionally, the virus had been passaged four times in C6/36 cells prior to the sequential and alternating cell culture passaging experiments. Although we purposefully used a local isolate with a low passage history, we cannot dismiss the possibility that the four initial passages in C6/36 cells had a differential effect on viral infectivity in the diverse cell types used in the studies described in this report.

## Conclusions

Despite the limitations of our experimental design, we provide evidence that the passage history of ZIKV in cell culture can influence the outcome of vector competence experiments, with mosquito cell-adapted ZIKV exhibiting greater fitness in its natural vector compared to its vertebrate cell-adapted and alternate-passaged counterparts. No statistical differences were observed among the infection, dissemination and transmission rates of the input and mosquito cell-adapted viruses in *Ae. aegypti* suggesting that infectivity was at best maintained by sequential passage in mosquito cells while the vertebrate cell-adapted and alternate-passaged viruses apparently lost infectivity. Studies on the adaptation of arboviruses to local mosquito populations are important because they provide insight into their transmissibility, vector and host range and epidemic potential.

## Supplementary Information

**Additional file 1: Table S1.** Comparison of genomic RNA copy numbers in organs and other body parts of *Ae. aegypti* challenged with input Zika virus (ZIKV-I). **Table S2.** Comparison of genomic RNA copy numbers in organs and other body parts of *Ae. aegypti* challenged with C6/36 cell-adapted Zika virus (ZIKV-6C).

## Data Availability

The nucleotide sequences obtained during the current study are available in GenBank under the accession numbers MT507047–MT507050.
