# Supplementary material for: Infection, dissemination, and transmission efficiencies of Zika virus in Aedes aegypti after serial passage in mosquito or mammalian cell lines or alternating passage in both cell types
Source: Parasit Vectors. 2021 May 18;14:261. doi: 10.1186/s13071-021-04726-1 (PMC8130322; doi:10.1186/s13071-021-04726-1)
Supplement: Supplementary file 1 — Additional file 1: Table S1. Comparison of genomic RNA copy numbers in organs and other body parts of Ae. aegypti challenged with input Zika virus (ZIKV-I). Table S2. Comparison of genomic RNA copy numbers in organs and other body parts of Ae. aegypti challenged with C6/36 cell-adapted Zika virus (ZIKV-6C). [file 13071_2021_4726_MOESM1_ESM.docx]

Infection, dissemination, and transmission efficiencies of Zika virus in *Aedes aegypti* after serial passage in mosquito or mammalian cell lines or alternating passage in both cell types.

Lourdes G. Talavera-Aguilar^1^, Reyes A. Murrieta^2^, Sungmin Kiem^3^, Rosa C. Cetina-Trejo^1^, Carlos M. Baak-Baak^1^, Gregory D. Ebel^2^, Bradley J. Blitvich^4^, Carlos Machain-Williams^1^.

^1^Laboratorio de Arbovirología, Centro de Investigaciones Regionales “Dr. Hideyo Noguchi”, Universidad Autónoma de Yucatán, Mérida, México; ^2^Department of Microbiology, Immunology and Pathology, College of Veterinary Medicine and Biomedical Sciences, Colorado State University, Fort Collins, CO, USA; ^3^Department of Infectious Diseases in Internal Medicine, Sejong Chungnam National University Hospital, School of Medicine, Chungnam National University, Sejong, Korea; ^4^Department of Veterinary Microbiology and Preventive Medicine, College of Veterinary Medicine, Iowa State University, Ames, Iowa, USA

Corresponding Author: Carlos Machain-Williams, Laboratorio de Arbovirología, Centro de Investigaciones Regionales “Dr. Hideyo Noguchi”, Universidad Autónoma de Yucatán, Mérida, México; e-mail: carlos.machain@correo.uady.mx

Key words: Zika virus, flavivirus, *Aedes aegypti*, adaptive mutations, vectorial competence

**Table S1.** Comparison of genomic RNA copy numbers in organs and other body parts of *Ae. aegypti* challenged with input Zika virus (ZIKV-I).

| Organ | Time  (days post challenge) | Median  (RNA copies/µL) | *X^2^* | Df | *P*-value |
| --- | --- | --- | --- | --- | --- |
| Malpighian tubules | 7 | 1,514 | 9.33 | 2 | 0.009 |
|  | 14 | 3,946 |  |  |  |
|  | 21 | 487,072 |  |  |  |
| Midgut | 3 | 5,025,875 | 19.84 | 4 | 0.001 |
|  | 5 | 426,055 |  |  |  |
|  | 7 | 143,492 |  |  |  |
|  | 14 | 900,854 |  |  |  |
|  | 21 | 1,405,356 |  |  |  |
| Ovaries | 7 | 220 | 14.00 | 2 | 0.001 |
|  | 14 | 3,002 |  |  |  |
|  | 21 | 148,841 |  |  |  |
| Salivary glands | 14 | 275,125 | 6.54 | 1 | 0.011 |
|  | 21 | 485,716 |  |  |  |
| Wings/Legs | 7 | 6,990 | 9.75 | 2 | 0.008 |
|  | 14 | 40,469 |  |  |  |
|  | 21 | 103,994 |  |  |  |

Friedman test (p≤0.05)

**Table S2.** Comparison of genomic RNA copy numbers in organs and other body parts of *Ae. aegypti* challenged with C6/36 cell-adapted Zika virus (ZIKV-6C).

| Organ | Time  (days post challenge) | Median  (RNA copies/µL) | *X^2^* | Df | *P*-value |
| --- | --- | --- | --- | --- | --- |
| Malpighian tubules | 7 | 21,80 | 6.22 | 2 | 0.045 |
|  | 14 | 17,110 |  |  |  |
|  | 21 | 106,189 |  |  |  |
| Midgut | 3 | 111,996 | 41.88 | 4 | 0.000 |
|  | 5 | 1,523,713 |  |  |  |
|  | 7 | 1,508,760 |  |  |  |
|  | 14 | 947,204 |  |  |  |
|  | 21 | 2,040,583 |  |  |  |
| Ovaries | 7 | 591 | 9.33 | 2 | 0.009 |
|  | 14 | 9,743 |  |  |  |
|  | 21 | 446,591 |  |  |  |
| Salivary glands | 7 | 966 | 10.00 | 2 | 0.007 |
|  | 14 | 250,854 |  |  |  |
|  | 21 | 871,801 |  |  |  |
| Wings/Legs | 7 | 6,206 | 18.20 | 2 | 0.000 |
|  | 14 | 23,287 |  |  |  |
|  | 21 | 213,936 |  |  |  |

Friedman test (p≤0.05)
